# Genetic Diversity of the Symbiotic Fungus *Epichloë festucae* in Naturally Occurring Host Grass Populations

**DOI:** 10.3389/fmicb.2021.756991

**Published:** 2021-12-03

**Authors:** Maria von Cräutlein, Marjo Helander, Helena Korpelainen, Päivi Helena Leinonen, Beatriz R. Vázquez de Aldana, Carolyn Anne Young, Iñigo Zabalgogeazcoa, Kari Saikkonen

**Affiliations:** ^1^Department of Agricultural Sciences, Viikki Plant Science Centre, University of Helsinki, Helsinki, Finland; ^2^Management and Production of Renewable Resources, Natural Resources Institute Finland (Luke), Helsinki, Finland; ^3^Biodiversity Unit, University of Turku, Turku, Finland; ^4^Department of Biology, University of Turku, Turku, Finland; ^5^Institute of Natural Resources and Agrobiology of Salamanca, Spanish National Research Council (CSIC), Salamanca, Spain; ^6^Noble Research Institute, Ardmore, OK, United States; ^7^Management and Production of Renewable Resources, Natural Resources Institute Finland (Luke), Turku, Finland

**Keywords:** alkaloid production, *Epichloë festucae*, *Festuca rubra*, ergot alkaloid, indole-diterpene, pyrrolpyrazine, reproductive modes, genetic population structure

## Abstract

*Epichloë* festucae is a common symbiont of the perennial and widely distributed cool season grass, *Festuca rubra*. The symbiosis is highly integrated involving systemic growth of the fungus throughout above-ground host parts and vertical transmission from plant to its offspring *via* host seeds. However, the nature of symbiosis is labile ranging from antagonistic to mutualistic depending on prevailing selection pressures. Both the loss of fungus in the maternal host lineage and horizontal transmission through sexual spores within the host population may partly explain the detected variation in symbiosis in wild grass populations. Epichloë species are commonly considered as pathogens when they produce sexual spores and partly castrate their host plant. This is the pathogenic end of the continuum from antagonistic to mutualistic interactions. Here we examined the population genetic structure of *E. festucae* to reveal the gene flow, importance of reproduction modes, and alkaloid potential of the symbiotic fungus in Europe. *Epichl*oë-species are highly dependent on the host in survival and reproduction whilst benefits to the host are largely linked to defensive mutualism attributable to fungal-origin bioactive alkaloids that negatively affect vertebrate and/or invertebrate herbivores. We detected decreased genetic diversity in previously glaciated areas compared to non-glaciated regions during the last glacial maximum period and found three major genetic clusters in *E. festucae* populations: southern, northeastern and northwestern Europe. Sexual reproduction may have a higher role than expected in Spanish *E. festucae* populations due to the predominance of unique genotypes and presence of both mating types in the region. In contrast, asexual reproduction *via* host seeds predominates in the Faroe Island and Finland in northern Europe due to the presence of biased mating-type ratios and large dominant genotypes in the *E. festucae* populations within the region. A substantially larger variation of alkaloid genotypes was observed in the fungal populations than expected, although the variability of the alkaloid genotypes within populations is considerably lower in northern than Spanish populations in southern Europe. *E. festucae* populations consist of different combinations of alkaloid classes from the gene clusters of ergot alkaloid and indole-terpenes, and from pyrrolopyrazine alkaloid gene. We suggest that the postglacial distribution history of the host grass, prevailing reproduction strategies of *E. festucae*, and local selection pressures likely explain a large part of the genetic variation observed in fungal populations among geographic regions. The identified alkaloid genotypes can be used by turfgrass breeders to improve resistance against herbivores in red fescue varieties and to develop new sustainable cultivars in Europe.

## Introduction

Microbes are ubiquitous and involved in all biogeochemical processes supporting the evolutionary trajectories from the origin of life. Earliest free-living microbes enabled terrestrial life on Earth by producing atmospheric oxygen (Wellman and Strother, 2015), and since then reciprocal adaptations and counter adaptations between microbes and higher organisms have driven adaptive radiation of species (Janzen, 1980; Thompson, 1994, 2005; Saikkonen et al., 2020). As adaptive capacity of microbes is unparalleled, microbes still sustain and organize biodiversity globally. For example, plant symbiotic bacteria and fungi are vital for host plant fitness during the primary succession as well as in hostile and extreme environments (Zilber-Rosenberg and Rosenberg, 2008; Nissinen et al., 2012; Kumar et al., 2017). Many of these symbiotic interactions are mutually beneficial and characterized by evolutionary outcomes such as species-specificity and vertical transmission of the microbial partner from host plant to its offspring, which will in turn select for benign association. However, vertically transmitted microbes could have a greater chance of destabilizing or constraining the symbiosis because it is often associated with the loss of contagious spread and the independent phase of the life cycle. Loss of contagious spread by sexual spores results in genetic host specificity, decreased sexual reproduction and recombination potential, and increased genetic uniformity of the microbe (Frank, 1994, 1996a,b; Nowak et al., 1994; Doebeli and Knowlton, 1998; Herre et al., 1999; Saikkonen et al., 1999, 2004). Thus, interactions between hereditary microbes and plants are context dependent and ranging from antagonistic to mutualistic (Saikkonen et al., 1998, 2010a).

Here we examine the genetic structure of fungal symbiont, *Epichlo*ë *festucae* Leuchtmann, Schardl, and Siegel (Clavicipitaceae, Hypocreales, Ascomycota), commonly associated with the widely distributed cool-season perennial grass, *Festuca rubra* L. (Poaceae, subfamily Pooideae) (Dirihan et al., 2016; von Cräutlein et al., 2019). Transmission and reproductive modes of *E. festucae* provide unique opportunities to study how life history traits of the fungal partner may translate into adaptable genetics determining the ecology of symbiotum. A single filamentous *E. festucae* genotype typically forms systemic and asymptomatic association throughout the above ground parts of the host plant, including the developing seeds (Clay and Schardl, 2002; Tadych et al., 2014). In addition to asexual distribution *via* vertical transmission to the seeds, *E. festucae* can occasionally spread horizontally by sexual spores within grass populations (Clay and Schardl, 2002). A similar mixed strategy involving both sexual and asexual reproduction is characteristic of numerous haploid fungal symbiotic microorganisms (Milgroom, 1996, 1997). As *E. festucae* is a heterothallic obligate out-crosser with two different mating types, fertilization requires dispersal of spermatia (male gametes) to an unfertilized fruiting body, a stroma, of opposite mating type vectored by phylogenetically distinct clade of anthomyiid flies (*Botanophila* ssp.) (Leuchtmann and Michelsen, 2016). This allows sexual crossing and perithecial development on the stroma surface (Bultman and White, 1988; Bultman and Leuchtmann, 2003). The stroma envelops an inflorescence and prevents seed development of the enclosed florets, causing a syndrome known as a choke disease (White et al., 1991; Schardl, 2010; Tadych et al., 2014). A mature stroma bears numerous perithecia with elongated asci, which produce filiform wind-dispersed haploid ascospores that mediate transmission to new hosts by infecting the host ovule (White, 1988). The choking stromata in *F. rubra* have been observed only in few flowering stems in Spain (Zabalgogeazcoa et al., 1999), and a stroma is usually formed in few tillers of an individual host resulting in simultaneous asexual and sexual reproduction efforts of the fungus in a grass population (Schardl, 2001). Thus, *E. festucae* has only limited recombination potential and opportunities of contagious spread linking its fitness tightly to the fitness of the host grass and aligning the coevolution of the interaction toward mutually beneficial cooperation (Clay, 1998; Saikkonen et al., 1998, 2004, 2016). However, empirical evidence has revealed that the interaction between *Epichloë*-species and their host grass, similarly to all biological interactions, are context dependent and labile but can be mutualistic in some environments (Saikkonen et al., 1998; Cheplick and Faeth, 2009; Decunta et al., 2021).

*Epichloë*-species are highly dependent on the host grasses whilst the benefits from the interaction remains conditional to the host. In most cases the specific Epichloë species or isolate is either directly or indirectly linked to defensive mutualism attributable to alkaloids (Saikkonen et al., 2006, 2010a; Clay, 2009). Ecological consequences, however, may vary depending on the alkaloid profile of symbiota determined by the genotype of the fungus and prevailing environmental conditions (Morse et al., 2007; Schardl et al., 2012, 2013a; Saikkonen et al., 2013, 2016). The alkaloids providing defense against herbivores fall into four classes - ergot alkaloids, indole-diterpenes, lolines and pyrrolopyrazines as peramine - that differ in deterrence and toxicity to invertebrate and vertebrate herbivores (Schardl et al., 2012, 2013b,c; Berry et al., 2019). Peramine can deter insects, lolines are insecticidal whereas ergot alkaloids and indole-diterpenes are well known for their toxicity to vertebrate grazers (Clay et al., 1985; Tanaka et al., 2005; Schardl et al., 2006, 2007, 2013b,c; Crawford et al., 2010; Berry et al., 2015; Saikkonen et al., 2016).

In concordance with the reputed context-dependency between hereditary microbes and their host plants, our earlier studies have revealed that the natural populations of *F. rubra* are formed by structured mosaics of *Epichlo*ë*-*free and *Epichlo*ë*-*symbiotic grass individuals (Zabalgogeazcoa et al., 1999, 2006; Saikkonen et al., 2000; Arroyo Garcia et al., 2002; Wäli et al., 2007; Dirihan et al., 2016; Leinonen et al., 2019; von Cräutlein et al., 2019). In addition to postglacial colonization history of the species and their local coadaptation to prevailing selection forces, highly integrated morphological and life history traits seem to determine the geographic variation in the frequencies of *Epichlo*ë*-*symbiotic grasses. Numerous empirical studies, reviews and meta-analyses have demonstrated that herbivory best explains high *Epichloë* frequencies in grass populations. We have been sampling and monitoring *F. rubra* populations in relation to occurrences of *E. festucae* in Spain, Switzerland, Greenland, Faroe Islands, Iceland, Norway and Finland from south to north during the last 20 years (Dirihan et al., 2016). Herbivory appears to be important force promoting the symbiosis in our study populations in Faroe Islands, Northern Finland and Spain as the highest *Epichlo*ë frequencies are subjected to heavy grazing pressure by sheep, reindeer and cattle, respectively. Thus, considerably lower numbers of *E. festucae* infections has been found in all the other studied regions without the presence of intense herbivory. Yet an unanswered question is whether the distribution of *E. festucae* is primarily determined by herbivory selection operating on the symbiotum rather than the distribution history of the host grass and founder effect, i.e., coincidental distribution history of *E. festucae*-symbiontic host grasses.

In this study, we examine genetic population structure and importance of reproduction modes, and predict alkaloid production potential of *E. festucae* across Europe using nuclear microsatellite (SSR) markers as well as mating type and alkaloid gene markers. Nuclear microsatellite markers enable us to make inferences about population structure, gene flow and genetic drift based on the patterns of genetic diversity within and among populations and regions. Moreover, SSR and mating type gene markers provide estimates of recombination potential and reproduction modes (asexual vs. sexual) based on the genotype frequencies, the structure of multilocus genotypes and mating type ratios (Milgroom, 1995). Alkaloid gene markers provide insights into ecological importance of alkaloid production in the studied populations. Accordingly, we predict that geographic variation and population differentiation detected should be structured and resemble each other if *E. festucae* is primarily spread *via* host grass seeds and herbivory defines recent and present phenotypic selection on the symbiotum. Furthermore, we hypothesize that genetic diversity should decrease toward the edges of *E. festucae* range in Europe due to potential genetic drift and strong selection. We also expect to detect the highest genetic diversity near areas that remained ice-free and in glacial refugias during the last glacial maximum period, as detected in the host grass *F. rubra* populations (van Zijll de Jong et al., 2008; von Cräutlein et al., 2019).

## Materials and Methods

### Plant Material, Fungal Isolation, and DNA Extraction

The plants used in this study were originally collected as a part of research examining the occurrence and ecological importance of *Epichloë festucae* in wild populations of *Festuca rubra* L. s.l. across Europe in 2011 (Dirihan et al., 2016; Leinonen et al., 2019; von Cräutlein et al., 2019; Saikkonen et al., 2020; Vázquez de Aldana et al., 2020). The initial plants were split and a copy of each individual was maintained in pots with a mixture of peat and sand in the greenhouses at the Ruisalo Botanical Garden of Turku University. Splitting grass plants is a common way of generating identical genetic copies of both the host and the endophyte as the endophyte systemically infects the above ground plant tissue. The initial plants were tested for endophyte infection with methods described in Dirihan et al. (2016). In this study, we focused on three European regions of the host distribution extreme range with the highest occurrence of *E. festucae* infections (Dirihan et al., 2016). We examined a total of 240 individual *Epichloë*-infected plants originating from 15 natural populations from six islands in the Faroe Islands (*n* = 71), from six populations located in two different habitat types, meadows (*n* = 73) and riverbanks (*n* = 30), in Finland, and from three populations located in two different habitat types, Mediterranean oak forest (*n* = 22) and semiarid oak grassland (*n* = 45), in Spain (*n* = 66). More detailed information on the plants, occurrence of associated *Epichloë*-fungus and collection sites, including population geographic locations, coordinates, altitudes, habitat features and estimate of grazing intensity, can be found in Table 1, Dirihan et al. (2016); von Cräutlein et al. (2019). The frequencies of *Epichloë*- individuals in the studied populations ranged from 5% to 81% and the number of individual isolates varied accordingly (Table 1; Dirihan et al., 2016).

**TABLE 1:** Collection sites, habitat features and occurrences of *Epichloë festucae* in host *Festuca rubra* populations based on Dirihan et al. (2016), von Cräutlein et al. (2019).

Population code	Geographic site	Population site	Geographic coordinates	Altitude (m a.s.l.)	Habitat	Grazing pressure	Endophyte infection%
FAS1	The Faroe Islands	Mykines	N 62°5′51″ W 7°40′56″	125	Meadow	High (sheep)	68
FAS2	The Faroe Islands	Vidoy	N 62°22′3″ W 6°32′32″	148	Meadow	High (sheep)	44
FAS3	The Faroe Islands	Sandoy	N 61°50′11″ W 6°51′21″	69	Meadow	High (sheep)	21
FAS4	The Faroe Islands	Nolsoy	N 62°1′15″ W 6°41′8″	55	Meadow	High (sheep)	5
FAS5	The Faroe Islands	Vagar	N 62°6′59″ W 7°26′43″	246	Meadow	High (sheep)	25
FAS6	The Faroe Islands	Eysturoy	N 62°17′24″ W 7°2′10″	316	Meadow	High (sheep)	54
MS1K	Finland	Kevo 1	N 69°38′6″ E 27°5′1″	91	Meadow	High (reindeer)	56
MS2K	Finland	Kevo 2	N 69°43′56″ E 27°12′0″	85	Meadow	High (reindeer)	75
KS3	Finland	Kevo 3	N 69°45′32″ E 26°59′19″	107	Meadow	High (reindeer)	50
RBS1	Finland	Kevo 4	N 69°54′36″ E 27°1′48″	73	Riverbank	High (reindeer)	45
RBS2	Finland	Kevo 5	N 69°56′41″ E 26°43′22″	85	Riverbank	High (reindeer)	20
RBS3	Finland	Kevo 6	N 69°56′11″ E 26°27′45″	106	Riverbank	High (reindeer)	23
SPGD	Spain	Garganta de los Infiernos	N 40°12′1″ W 5°45′11″	768	Mediterranean oak forest	Medium (cattle, goat, sheep)	81
SPLV	Spain	Salamanca 1	N 40°56′20″ W 6°7′7″	863	Semiarid oak grassland, dehesas	High (cattle)	67
SPPOR	Spain	Salamanca 2	N 40°58′24″ W 5°57′34″	812	Semiarid oak grassland, dehesas	High (cattle)	59

*Epichloë festucae* was isolated from the plants in 2013 and 2014. Three leaves from each tiller were selected from pots and tillers were surface sterilized. A leaf was cut in five segments and inoculated on autoclaved Petri dishes containing 5% potato dextrose agar (PDA). Plates were stored at room temperature until mycelium emerged, after which a small sample of mycelium were transferred to a new PDA plate on a piece of sterilized cellophane. Total genomic DNA was extracted from pure cultures of mycelium growth using the E.Z.N.A Plant DNA Kit (Omega Bio-Tek, Norcross, GA, United States) according to the procedures described in von Cräutlein et al. (2014). The same DNA samples were used for the analyses based on the SSR mating type and alkaloid gene markers.

### Genetic Population Structure and Reproduction Modes

Genetic structure and the amount of clonality in the 240 *E. festucae* isolates were investigated using 14 polymorphic SSR markers, which were developed based on the searches for ≥10 mono- and dinucleotide repeats, and for ≥8 tri-, tetra-, penta-, and hexanucleotide repeats in the unplaced genomic scaffold sequences of *E. festucae* (for section “Materials and Methods” see von Cräutlein et al., 2014). The forward primers of each SSR primer pair were end-labeled with two different phosphoramidite fluorescent dyes, either HEX or 6-FAM. The samples were analyzed by multiplexing markers (2-4 primer pairs/reaction) with different labels and expected fragment sizes. Allele sizes ranged from 92 to 340 bp depending on the primer pairs (Supplementary Table 1). The details of PCR amplifications are described in von Cräutlein et al. (2014). Each genotyping plate included negative and positive controls and samples from several populations. The PCR products were run on an ABI 3130xl DNA Sequencer using GeneScan 500 ROX Size standard (Applied Biosystems) at the Institute of Biotechnology, University of Helsinki, Finland. Peak Scanner version 1 software were used (Applied Biosystems) to assign the allelic sizes of the amplified fragments. The detailed information on SSR markers, including e.g., names and locations of markers in the *E. festucae* chromosomes, are described in Supplementary Table 1 and whole SSR data set with host plant IDs in Supplementary Table 2. As *E. festucae* is haploid, the samples were expected, and did produce one allele per locus. However, in the rare exception where multiple alleles were observed in at least one SSR locus of Faroe Islands (five isolates), Finland (six isolates), and Spain (six isolates), the samples were not included in the study.

### Mating Type and Alkaloid Gene Variation

The genetic loci involved in alkaloid biosynthetic pathways essential for the production of ergot alkaloids (*EAS*), indole-diterpenes (*IDT*) and lolines (*LOL*) are complex gene clusters in *Epichloë* taxa, whereas the pyrrolopyrazine alkaloids (*PPZ*, previously referred as *PER*) production is dependent on the alleles of the *perA* gene (Tanaka et al., 2005; Schardl et al., 2012, 2013b; Berry et al., 2015, 2019). The genes encoding different alkaloid classes have recently been identified (Schardl et al., 2013b) allowing us to predict alkaloid production based on presence or absence of a key alkaloid genes within the pathway (Takach et al., 2012; Schardl et al., 2013b,c; Charlton et al., 2014; Takach and Young, 2014; Berry et al., 2015; Shymanovich et al., 2015; Vikuk et al., 2019). These genes are upregulated in planta (Young et al., 2006, 2015; Chujo and Scott, 2014) and the gene clusters are devoid of known pathway specific regulatory genes, unlike other fungal secondary metabolite clusters that often contain a gene that encodes a regulatory function in the form of pathway specific transcription factors (e.g., aflR required for aflatoxin production; Woloshuk et al., 1994).

The presence of selected key genes from the loci for alkaloid production and mating type idiomorphs (genes *mtAC* and *mtBA*) were examined in a total of 198 *E. festucae* isolates originating from six populations in the Faroe Islands (*n* = 60), six populations in Finland (*n* = 91) and three populations in Spain (*n* = 46) (Table 1).

A multiplex PCR method was used to determine the mating type (*A* or *B*) and key genes present at each alkaloid loci (Charlton et al., 2014). The primers including two additional primers for *IDT* genes (*idtK* and *idtF*) used for mating type and alkaloid gene profiling, expected product sizes and six different multiplex sets are described in Supplementary Material in Charlton et al. (2014). The PCR amplification methods are described in Charlton et al. (2014). PCR products were analyzed by gel electrophoresis on a 1.5% agarose gel and visualized with ethidium bromide by UV transillumination. The combination of two samples, MS2K-35 and SPGD-31 was positive for all examined alkaloid genes and used as a positive control on each PCR plate. The presence of a nonsense mutation and inframe stop codon in the first exon of *idtF* gene were determined in isolates that contained the *IDT* genes required for terpendole C or lolitrem B productions. The primers *idtF-M-F* (5′-GGGCCATCCTATCTTACAC-3′) and *idtF-M-R* (5′-ACGAAGCCTTGAATCCAC-3′) were designed based on the *idtF* gene sequences with and without the mutation (GenBank accession numbers: EU530694 and MF464362). The PCR product of each alkaloid gene locus was sequenced using the following methods: the PCR products were separated on a 1.2% agarose gel, extracted from the gel and purified with E.Z.N.A. Gel Extraction Kit (Omega, Bio-Tek). The purified PCR products were submitted to Macrogen Inc., for Sanger sequencing with both upstream and downstream primers. The obtained sequences were visualized and manually corrected using Chromas version 2.6.5 (2018). The sequence similarity searches were performed in GenBank using BLAST sequence analysis tool (NCBI). All sequences showed the closest match (100% similarity) with *E. festucae* and the alkaloid gene region in question. The sequences were submitted to the European Nucleotide Archive. Accession numbers are available in Supplementary Table 3 for the positive controls of the alkaloid genes and for the presence or absence of deletion in the *idtF* gene in the set of samples.

The presence or absence of a set of key alkaloid genes within the alkaloid class pathway detected here are described in Charlton et al. (2014). Ergovaline was expected to be produced, if five examined *EAS* genes (*dmaW, easC, easA, cloA*, and *lpsB*) produced PCR bands of the expected size, and chanoclavine was expected if only *dmaW* and *easC* were present. Peramine, a pyrrolpyrazine-1-one, was assumed to be produced, if all three markers including *perA5*′, *perAT2* and *perAR*, produced PCR bands, and if the reductase domain (*perA*R) was absent, then pyrrolpyrazine-1, 4-dione (PPZ-1 diones) were expected (Berry et al., 2019). Ergovaline and peramine chemotypes of some of the *F. rubra – E. festucae* symbionts in the present study (*n* = 27) determined by Vázquez de Aldana et al. (2020) were compared with the alkaloid gene profiles obtained in this study to confirm prediction of the alkaloid production. The first stable indole-diterpene intermediate, paspaline, is predicted if *idtG* and *idtQ* are both present. Isolates that can produce early pathway terpendoles, such as terpendoles E and I, also contain a functional *idtF*, and isolates that can produce late pathway terpendoles, such as terpendole C, contain *idtF* and *idtK* in addition to *idtG* and *idtQ.* Lolitrem B (LTB), the end product of IDT biosynthesis in *E. festucae*, was predicted to be produced, if in addition to the genes mentioned above also *idtJ* produced a PCR band. The prerequisite of the production of terpendole C and lolitrem B was also that the sequence of *idtF*-gene was functional, without the deletion in the first exon of the gene that causes an inframe stop codon (Young et al., 2009; Shi et al., 2017; Yi et al., 2018). In addition, presence of *idtP* gene was checked from our unpublished data in 109 of the *F. rubra – E. festucae* combinations in the present study. The host samples used in alkaloid gene, *idtF* mutation and *idtP*-gene detections are provided in Supplementary Table 4. Lolines were predicted to be produced, if examined *LOL* genes (*lolC, lolA, lolO*, and *lolP*) produced PCR bands.

The number of multilocus alkaloid gene genotypes (aMLG) based on the presence (1) or absence (0) of each key alkaloid gene was determined using haploid binary data and multilocus options within populations, across populations within regions and across the whole data set. For the alkaloid gene data set, pairwise PHi_*PT*_ values were used to estimate population pairwise differentiation levels within regions, two populations, FAS4 and RBS3, were excluded from the analysis, because of low numbers (<5) of isolates per population using the GenAlex version 6.5. (Peakall and Smouse, 2006, 2012).

### Statistical Analysis of Nuclear Microsatellite Markers

The number of multilocus SSR genotypes (nMLG), the number of expected genotypes based on rarefaction (eMLG) and random association among loci (indices of associations: I_*A*_ and r_*d*_; clone corrected data, 999 permutations) were computed for each population, across Finnish, Faroese and Spanish populations within regions and across the whole data set. Tests of random association among loci were not performed for the Faroese and Finnish populations due to lack of statistical power because of a low sample size after clone correction (Fincham and Day, 1963). Minimum spanning networks (MSN) using Bruvo’s distance were computed across all populations (Bruvo et al., 2004). The analyses were computed with R 3.5.1 (R Core Team, 2016) package *poppr* (v2.8.5.; Kamvar et al., 2014).

To study the genetic relationships of isolates, a Bayesian Analysis of Population Structure (BAPS) software, version 6.0, was used by applying a non-spatial mixture clustering analysis at individual level (sampling unit) with linked loci option (Corander and Tang, 2007; Corander et al., 2008) using multilocus SSR data set including all the isolates in order to represent the distribution of allele frequencies in this randomized sample set (*n* = 240). The partition of optimal K numbers, which refer to number of groups into which the SSR data can be clustered, was conducted by performing 150 iterations of K from 2 to 30, which resulted in the number of genetically diverged clusters in optimal partition to be 15 [log(marginal likelihood) value = −1901,6]. Fixed K model was used, because BAPS identified several small clusters (nine clusters with an average of 2.7 individuals). The number of clusters for the fixed K model was determined based on the uppermost hierarchical levels of genetic structure shown in a UPGMA tree based on the Kullback-Leibler divergence matrix. The individual level mixture clustering analysis with linked option was conducted using the fixed K mode with 150 iterations of *K* = 4.

Genetic diversity indices of the SSR were calculated with two data sets by using all isolates (*n* = 240) and using all unique MLGs within regions (*n* = 103) with haploid data option and are based on the numbers of allele frequencies at each locus. The percentage of polymorphic loci (P%), the average effective numbers of alleles (Ne), the average numbers of unique alleles (Np) and unbiased genetic diversity (uh) estimates were calculated for each locus (*n* = 14), over the entire sample set (*n* = 240), over the MLGs (*n* = 103), for each region (*n* = 3), for each population (*n* = 15) and for each genetic group obtained by BAPS analysis (*K* = 4). A principal coordinate analysis (PCoA) was used to plot the major patterns in SSR data sets based on the whole data set (*n* = 240) and separately for each region (Faroe, Finland, and Spain) without clone corrections in relation to the genetic similarities of the isolates using pairwise individual-by-individual haploid genetic distance matrixes. Tests for significance were run with 999 random permutations. Analyses were conducted using GenAlex v. 6.5.

A hierarchical analysis of molecular variance (AMOVA; Weir and Cockerham, 1984; Excoffier et al., 1992; Weir, 1996) was used to estimate the degree of differentiation among regions and populations, and pairwise Fst values were used to estimate population differentiation levels among populations, regions, habitats within region and genetic clusters (*K* = 4) in SSR data set (*n* = 240, all isolates included). Moreover, the degree of differentiation among regions were also estimated with the data set with unique MLGs (*n* = 103). The analysis was conducted using Arlequin software, version 3.5 (Excoffier and Lischer, 2010). The significance of the fixation indices was run with 999 non-parametric permutations.

## Results

### Population Structure

**FIGURE 1:**
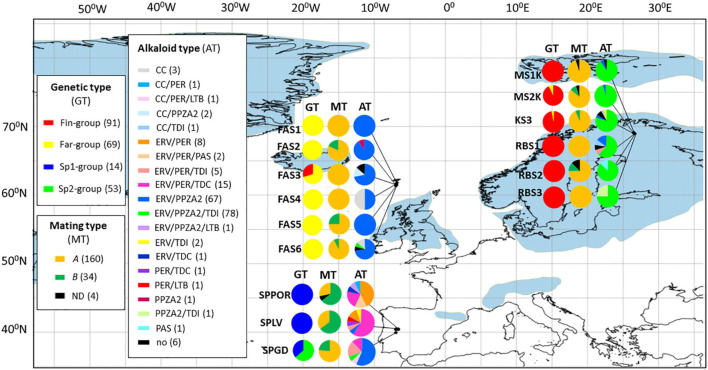
The frequencies of genetic (GT), mating (MT), and alkaloid types (AT) of *Epichloë festucae* isolates are visualized in the fifteen populations located in the Faroe Islands, Finland, and Spain. The genetic types are presented according to the Bayesian partition of the genetic groups (*K* = 4: *Fin*-, *Far*-, *Sp1-, or Sp2-group*), mating types (*A* or *B*) according to the presence of mating type genes (*mtAC*, *mtBA*, ND, not determined) and alkaloid types according to the predicted alkaloid production based on the alkaloid markers (in brackets are indicated the numbers of isolates within the alkaloid type). No = no alkaloid production predicted. Detailed information about alkaloid types are shown in Table 5. Blue regions indicate permanent ice cover during the last glacial period (25,000–15,000 years ago; Ray and Adams, 2001).

The Bayesian mixture analysis was conducted using fixed K mode of *K* = 4 [log (marginal likelihood) value = −2194.4]. At *K* = 4, the isolates are mainly distributed according to the geographical regions (Figure 1). *Fin*-group (*n* = 104) represents mainly Finnish isolates (*n* = 101) and three isolates from the Faroe Islands (FAS3). *Far*-group (*n* = 69) contains mainly Faroese isolates (*n* = 67) and two isolates from Finnish populations (KS3, RBS1). The Spanish isolates were partitioned in one large and one smaller genetic group. *Sp1*-group includes fourteen isolates from SPGD population. *Sp2*-group (*n* = 53) represents all isolates from SPLV (*n* = 23) and SPPOR (*n* = 21) but also eight isolates from SPGD and one isolate from the Faroe Islands (FAS6).

**FIGURE 2:**
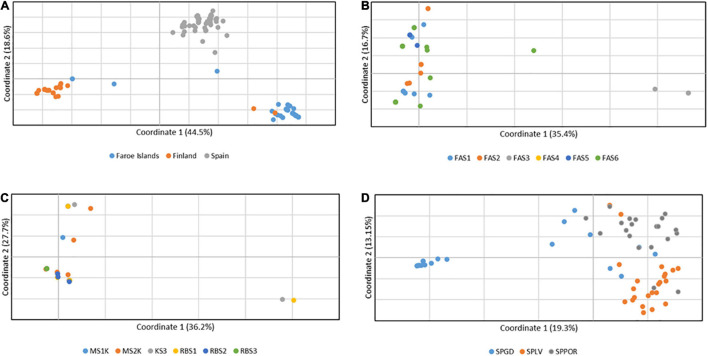
The results of principal coordinate analysis (PCoA) based on pairwise individual-by-individual haploid genetic distance matrixes of the nuclear microsatellite data **(A)** among all the Finnish, Faroese and Spanish *Epichloë festucae* isolates (*n* = 240), **(B)** among Faroese isolates (*n* = 71), **(C)** among Finnish isolates (*n* = 103), and **(D)** among Spanish isolates (*n* = 66). A clear geographic pattern of three groups corresponds to the genetic groups identified by Bayesian mixture analysis (*Far, Fin*, and *Sp*). The Spanish genetic groups, *Sp1* and *Sp2*, identified by Bayesian mixture analysis, are separated in the PcoA analysis among Spanish isolates.

Consistent with the results of Bayesian mixture analysis, a clear geographic pattern of three groups was detected among all the Finnish, Faroese and Spanish isolates (*n* = 240) according to the principal coordinate analyses based on pairwise individual-by-individual haploid genetic distance matrixes, in which the first and second axes explained 44.5% and 18.6% of the variation, respectively (Figure 2A). In Faroe Islands, three individuals differed from the main group, which was highly mixed with individuals from different populations, the first and second axes explained 35.4% and 16.7.% of the variation, respectively (Figure 2B). In Finland, two individuals differed from the main group, which was also mixed with individuals from different populations, the first and second axes explained 36.2% and 27.7% of the variation, respectively (Figure 2C). In Spain, individuals were distributed in one small and one large group and individuals of the larger groups were moderately mixed, the first and second axes explained 19.3% and 13.2% of the variation, respectively (Figure 2D).

### Genetic Diversity and Population Differentiation

Relatively low levels of genetic variation indices and numbers of private alleles were detected in most of the Finnish and Faroese populations, although the levels of genetic variation differed widely among the populations (Table 2). In contrast, relatively high levels of genetic variation indices and high numbers of private alleles were found in all Spanish populations (Table 2). Accordingly to the regions, genetic diversity estimates differed between the geographic locations of genetic groups (BAPS clusters) being highest in Spanish *Sp2*-group and clearly lower in *Far*- and *Fin*-groups (Table 2).

**TABLE 2:** Genotype and genetic diversity and indices of association by populations, by regions (in bold) and by genetic groups (Bayesian Analysis of Population Structure, *K* = 4) based on all the isolates of nuclear microsatellite data set (*n* = 240) in *Epichloë festucae*.

		Genotype diversity				Genetic diversity				Index of association		
	No. of isolates	No. of MLG	No. of eMLG	No. of unique MLG	No. of common MLG	*P* (%)	Ne	Np	uh	I_*A*_	r_*d*_	shared *p*-Value
**SSR**												
**by populations**												
FAS1	18	7	5	4	3	42.9	1.118	0.07	0.086	nt	nt	nt
FAS2	10	7	7	4	4	28.6	1.269	0	0.154	nt	nt	nt
FAS3	10	4	4	1	3	92.9	1.668	0.07	0.427	nt	nt	nt
FAS4	5	2	2	0	2	7.1	1.034	0	0.029	nt	nt	nt
FAS5	9	5	5	1	4	21.4	1.117	0	0.081	nt	nt	nt
FAS6	19	8	5.4	5	3	71.4	1.231	0.29	0.152	nt	nt	nt
**Faroe Islands**	**71**	**23**	**22**	**15**	**8**	**100**	**1.34 (1.69)**	**0.57**	**0.197 (0.346)**	**4.65**	**0.364**	**0.001**
MS1K	21	4	3	2	2	14.3	1.080	0.07	0.045	nt	nt	nt
MS2K	25	7	3.9	5	2	35.7	1.068	0.14	0.051	nt	nt	nt
KS3	27	8	4.2	4	4	85.7	1.168	0.14	0.128	nt	nt	nt
RBS1	14	5	4.1	1	4	85.7	1.200	0	0.168	nt	nt	nt
RBS2	10	4	4	3	1	21.4	1.065	0.14	0.054	nt	nt	nt
RBS3	6	1	1	0	1	0	1.000	0	0	nt	nt	nt
**Finland**	**103**	**19**	**13.4**	**15**	**4**	**92.9**	**1.11 (1.47)**	**0.50**	**0.085 (0.298)**	**4.08**	**0.364**	**0.001**
SPGD	22	18	9.1	15	3	100	2.372	1.36	0.473	4.544	0.359	0.001
SPLV	23	23	10	23	0	100	2.963	1.36	0.632	0.967	0.075	0.001
SPPOR	21	20	9.8	19	1	100	2.831	1.00	0.566	1.273	0.100	0.001
**Spain**	**66**	**61**	**61**	**57**	**4**	**100**	**3.75 (3.85)**	**6.43**	**0.682 (0.690)**	**1.05**	**0.083**	**0.001**
TOTAL	240	103	-	86	17	100	3.33	10.71	0.676	3.43	0.270	0.001
**by genetic groups**												
*Far*	69	22	7.9	14	8	71.4	1.22 (1.40)	0.43	0.124 (0.206)	0.329	0.043	0.002
*Fin*	104	19	4.7	14	5	92.9	1.09 (1.30)	0.79	0.068 (0.204)	0.624	0.065	0.001
*Sp1*	14	10	10	7	3	35.7	1.41 (1.49)	0.57	0.133 (0.154)	nt	nt	nt
*Sp2*	53	52	13.9	51	1	100	3.99 (3.98)	5.64	0.688 (0.692)	1.033	0.082	0.001

*In brackets are shown the genetic diversity indices (Ne, uh) calculated in clone corrected data set for Faroe Islands (MLG *n* = 23), Finland (MLG *n* = 19) and Spain (MLG *n* = 61) and for genetic groups (BAPS, *K* = 4). MLG, multilocus genotypes; eMLG, expected MLG based on rarefaction; P%, percentage of polymorphic loci; Ne, average effective numbers of alleles; Np, average numbers of unique alleles; uh, unbiased genetic diversity; I_*A*_ and rd: Indices of association: clone corrected data; nt, not tested because of too low number of MLGs for the analysis.Occur only in one region.*

Overall using all the isolates (*n* = 240), the AMOVA analysis estimated that 66.6% of the genetic variation occurs among regions, 36.1% within populations and only 7.3% among populations within regions indicating high differentiation among the regions and low differentiation among the populations (Table 3). Based on the data set of unique MLGs (*n* = 103) for the regions, 44% of the genetic variation occurs among populations and 56% within populations (Table 3).

**TABLE 3:** Results of analysis of molecular variance analysis for the whole data set, the geographical regions and genetic groups (Bayesian Analysis of Population Structure, *K* = 4) calculated separately using all the isolates (*n* = 240) and using unique MLGs (*n* = 103) in *Epichloë festucae*.

Origin	d.f.	Sum of Squares	Variance components	Variance (%)	*p*
**Geographical regions, including all isolates**					
Whole data set					
Among regions	2	1326.4	4.115	66.6	<0.001
Among populations within regions	12	184.7	0.451	7.3	<0.001
Within populations	465	749.6	1.612	36.1	<0.001
Faroe Islands					
Among populations	5	49.6	0.388	26.9	<0.001
Within populations	136	143.6	1.056	73.1	<0.001
Finland					
Among populations	5	7.3	0.027	4.5	0.192
Within populations	200	113.6	0.567	95.5	<0.001
Spain					
Among populations	2	127.8	1.366	26.4	<0.001
Within populations	129	492.4	3.817	73.6	<0.001
**Georgaphical regions, including unique MLGs**					
Among regions	2	350.6	2.646	44.0	<0.001
Within populations	203	761.7	3.752	56.0	<0.001
SSR markers					
**Genetic groups, including all isolates**					
Among groups	3	1518.9	4.657	74.9	<0.001
Within groups	476	741.8	1.558	25.1	<0.001
**Genetic groups, including unique MLGs**					
Among groups	3	486.6	3.532	53.3	<0.001
Within groups	202	625.8	3.098	46.7	<0.001

In the Faroe Islands (using all the Faroese isolates), most of the variation (73.1%) was detected within populations and moderate levels of genetic differentiation (26.9%) among the six populations (Table 3). The population pairwise Fst values varied from 0.004 to 0.447 being on average 0.25 ± 0.14 and significant differentiation levels were found among 67% of the population pairs (*p* < 0.05; *n* = 10; Supplementary Table 5A).

In Finland (using all the Finnish isolates), majority of the variation (95.5%) was within populations and only low level (4.5%) genetic differentiation was found among the six populations (Table 3). No significant differentiation was detected between the populations located in meadow (*n* = 3) and riverbank (*n* = 3) habitats (Fst = 0.067, *p* = 0.175, see Table 1). The population pairwise Fst values varied from 0.011 to 0.202 being on average 0.065 ± 0.060 and 20% of population pairs showed significant differentiation between populations (*p* < 0.05; *n* = 3; Supplementary Table 5A).

In Spain (using all the Spanish isolates), most of the variation (73.6%) was detected within populations and moderate level genetic differentiation (26.4%) observed among the three populations (Table 3). A significant differentiation was detected between the populations located in Mediterranean forest (SPGD) and dehesa grassland (SPLV and SPPOR) habitats (Fst = 0.295, *p* < 0.001, see Table 1). The population pairwise Fst values are shown in Supplementary Table 5A.

Among the genetic groups (*K* = 4, including all the isolates, *n* = 240), AMOVA analysis revealed that 75% of the genetic variation is distributed among the genetic groups and 25% within clusters indicating very high genetic differentiation among the groups (Table 3). Using the data set of unique MLGs for the region (*n* = 103), 53.3% of the genetic variation occurs among populations and 46.7% within populations (Table 3). The pairwise Fst values of genetic groups are shown in the data sets with all the isolates and with MLGs in Supplementary Table 5B.

### Reproduction Modes

**FIGURE 3:**
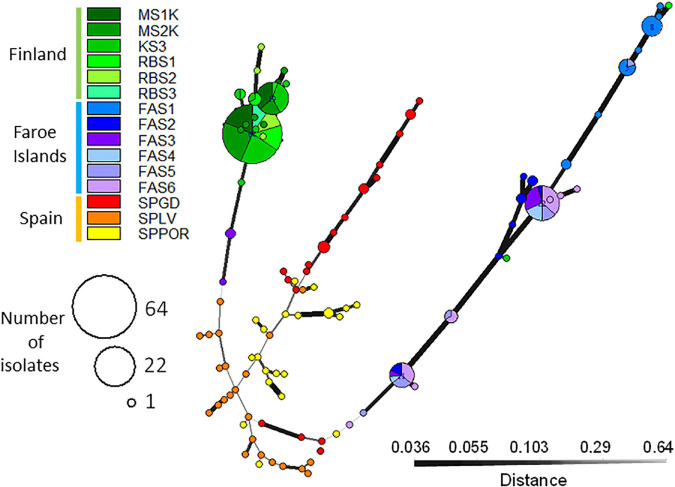
Minimum spanning networks visualize the genotype size (nMLGs) and the genetic distance among *Epichloë festucae* isolates from the populations located in Finland, the Faroe Islands and Spain. Each node represents a unique multilocus genotype and edge widths and shading represents relatedness according to Bruvo’s distance, edge length is arbitrary (Kamvar et al., 2014). The largest common nMLG includes 64 Finnish isolates and second largest nMLG 22 Faroese isolates. Single MLGs predominated among Spanish isolates and only three common nMLGs occurred in the region. Faroe Islands: FAS1, FAS2, FAS3, FAS4, FAS5, FAS6; Finland: MS1K, MS2K, KS3, RBS1, RBS2, RBS3; Spain: SPGD, SPLV, SPPOR.

The clonal structure of the isolates according to populations within regions is visualized in Figure 3. In total, 103 fungal multilocus nuclear genotypes (nMLG) were observed in the SSR data set (*n* = 240) in fifteen populations collected from Spain, the Faroe Islands and Finland (Table 2). High frequencies (92.4%) of unique nMLGs and eMLGs occurred in the Spanish populations and only four common genotypes were present in two populations, of which three nMLGs in Mediterranean oak population (SPGD) (Table 2). In the Faroe Islands, fifteen (65.2%) nMLGs were unique in the region and eight common nMLGs consist of 78.9% of the isolates (*n* = 56) located in different Faroese populations, three largest clone sizes are 22,11 and 8 isolates/nMLG. In Finland, fifteen (78.9%) nMLGs were unique in the region and four common nMLGs consist of 85.4% of the isolates (*n* = 88) located in different Finnish populations, two largest clone sizes are 64 and 19 isolates/nMLG.

The indices of associations (I_*A*_ and r_*d*_) were utilized to estimate the relative importance of reproduction types, i.e., either sexual or asexual reproduction predominated in the regions (Kamvar et al., 2014). Significant linkage disequilibrium was estimated in Finland, the Faroe Islands and Spain and separately in each Spanish population suggesting linked loci and predominance of asexual reproduction in all three regions, although indices of association were clearly lower in Spain than in Finland and the Faroe Islands (Table 2).

The frequencies of mating types according to the populations and genetic groups (*K* = 4) are shown in Figure 1 and Table 4. The ratio of mating types *A* and *B* was 0.92, therefore the proportions of each mating type are close to equal among populations in Spain. In SPLV and SPPOR populations most of the isolates were of mating type B, whereas most of SPGD isolates were of mating type *A*. In contrast, majority of the isolates in Finland and the Faroe Islands were of mating type *A*, although both mating types were present in half of the populations in both Finland and Faroe Islands. Mating type genes were not detected in four of the isolates. Based on the genetic groups (*K* = 4) *Fin*-, *Far*-, and *Sp2* -groups shared the mating types A and B, but only mating type A occurred in *Sp1*-group (Table 4).

**TABLE 4:** Frequencies of mating types *A* and *B* by populations (*n* = 198), by regions (in bold) and by genetic groups (Bayesian Analysis of Population Structure, *n* = 188) in *Epichloë festucae* in natural *Festuca rubra* populations.

		Mating types			
	No. of isolates	(*A*) *n* (%)	(*B*) *n* (%)	Ratio A:B	ND^*a*^ n (%)
by population					
FAS1	17	17 (100)	–	–	–
FAS2	11	9 (81.8)	2 (18.2)	4.5	–
FAS3	7	7 (100)	–	–	–
FAS4	2	2 (100)	–	–	–
FAS5	8	6 (75)	2 (25)	3.0	–
FAS6	15	14 (93.3)	1 (6.7)	14.0	–
**Faroe Islands**	**60**	**55 (91.7)**	**5 (8.3)**	**11.0**	–
MS1K	20	19 (95)	–	–	1 (5)
MS2K	21	18 (85.7)	2 (9.5)	9	1 (4.8)
KS3	25	23 (92)	2 (8.0)	11.5	–
RBS1	13	13 (100)	–	–	–
RBS2	8	6 (75)	1 (12.5)	6	1 (12.5)
RBS3	4	4 (100)	–	–	–
**Finland**	**91**	**83 (91.2)**	**5 (5.5)**	**28.3**	**3 (3.3)**
SPGD	16	12 (75)	4 (25)	3.3	–
SPLV	17	6 (35.3)	11 (64.7)	0.5	–
SPPOR	14	4 (28.6)	9 (64.3)	0.4	1 (7.1)
**Spain**	**47**	**22 (46.8)**	**24 (51.1)**	**0.9**	**1 (2.1)**
TOTAL	198	160	34	4.7	4
by genetic group					
*Far*	58	54 (93.1)	4 (6.9)	13.5	–
*Fin*	84	79 (94)	3 (3.6)	26.3	2 (2.4)
*Sp1*	9	9 (100)	0	–	–
*Sp2*	37	12 (32.4)	24 (64.9)	0.5	1 (2.7)
TOTAL	188	154 (81.9)	31 (16.5)	5.0	3 (1.6)

*^*a*^ND = not detected.*

**TABLE 5:** Alkaloid genotypes (aMLGs) of *Epichloë festucae* in the natural host populations of *Festuca rubra* in the Faroe Islands, Finland and Spain.

		No. of isolates within genotype			Ergot alkaloid (*EAS*) genes^*a*^					Peramine (*PER*) domains^*a*^			Indole-diterpenes (*IDT*) genes^*a*^							Alkaloid confirmed^*d*^	
aMLG	MT ratio A:B	Faroe	Finland	Spain	*dmaW*	*easC*	*easA*	*cloA*	*lpsB*	*perA5*	*perAT2*	*perAR*	*idtG*	*idtQ*	*idtP* ^*b*^	*idtF*	*idtK*	*idtJ*	Predicted chemotype^*c*^	*ERV*	*PER^*f*^*
gt-1	B	1			+	–	–	–	+	+	+	–	+	–	nt	–	–	–	PPZA2	nt	nt
gt-2	A	1			+	+	+	+	–	+	+	–	–	–	nt	–	–	–	CC/PPZA2	nt	nt
gt-3	A	1			+	–	–	–	–	–	–	–	+	–	–	–	–	–		nt	nt
gt-4	A	1			+	+	+	+	+	+	+	–	–	–	nt	–	+	–	ERV/PPZA2	nt	nt
gt-5	B	1			–	–	–	–	–	+	+	–	–	–	–	+	+	–	PPZA2	nt	nt
gt-8	A		1		+	+	+	–	+	–	+	–	+	+	nt	ψ^*e*^	+	–	CC/TDI	nt	nt
gt-9	B		1		+	+	+	+	+	–	+	–	+	+	+	ψ^*e*^	+	–	ERV/TDI	nt	nt
gt-10	B		1		–	–	–	–	+	–	+	–	–	–	+	–	–	–		nt	nt
gt-11	A		1		+	+	+	+	–	+	+	–	+	–	nt	+	+	–	CC/PPZA2	nt	nt
gt-12	A		1		+	–	–	+	–	–	+	–	+	–	nt	+	+	–		nt	nt
gt-13	nd		1		+	–	–	–	–	–	+	–	+	–	+	+	+	–	PAS	nt	nt
gt-14	A		1		+	+	+	+	–	+	+	–	+	+	nt	–	–	–	PPZA2/TDI	nt	nt
gt-15	A			1	+	+	–	+	–	+	+	+	+	+	+	+	+	+	CC/PER/LTB	nt	nt
gt-16	B			1	+	+	+	+	+	+	+	+	+	+	+	ψ^*e*^	+	+	ERV/PER/TDI	no	yes
gt-17	B			1	+	+	+	+	+	+	–	+	+	+	nt	–	–	–	ERV/TDI	nt	nt
gt-18	A			1	+	–	+	–	+	+	+	+	+	+	+	+	+	+	PER/LTB	nt	nt
gt-19	B			1	–	–	–	–	–	+	+	+	+	+	+	+	+	–	PER/TDC	nt	nt
gt-20	B			1	+	+	+	+	+	+	+	+	–	+	+	ψ^*e*^	+	–	ERV/PER	nt	nt
gt-21	B			1	+	+	+	+	–	+	+	+	–	–	nt	–	–	–	CC/PER	no	yes
gt-22	B			1	+	+	+	+	+	+	–	–	+	+	+	+	+	–	ERV/TDC	nt	nt
gt-23	nd			1	+	+	+	+	+	+	+	+	+	–	–	–	–	–	ERV/PER	nt	nt
gt-24	B			1	+	+	+	+	+	+	+	–	+	+	+	+	+	+	ERV/PPZA2LTB	nt	nt
gt-25	A, nd		2		+	–	–	–	–	–	+	–	+	–	+	–	–	–	PAS	nt	nt
gt-26	3:0	3			+	+	+	–	+	–	+	–	–	–	–	–	–	–	CC	nt	nt
gt-27	32:0	30	2		+	+	+	+	+	+	+	–	–	–	–	–	–	–	ERV/PPZA2	yes	no
gt-28	4:0			4	+	+	+	+	+	+	+	+	–	–	–	–	–	–	ERV/PER	yes	yes
gt-29	7:0	6	1		+	+	+	+	+	+	+	–	–	–	–	+	+	–	ERV/PPZA2	yes	no
gt-30	4:0	4			+	+	+	+	+	+	+	–	–	+	–	–	–	–	ERV/PPZA2	nt	nt
gt-31	2:0			2	+	+	+	+	+	+	+	+	–	+	–	–	–	–	ERV/PER	no	yes
gt-32	2:0	2			+	+	+	+	+	+	+	–	–	+	–	+	+	–	ERV/PPZA2	nt	nt
gt-33	8:4	9		3	+	+	+	+	+	+	+	–	+	–	–	–	–	–	ERV/PPZA2	no	no
gt-34	2:0			2	+	+	+	+	+	+	+	–	+	–	nt	–	+	–	ERV/PPZA2	nt	nt
gt-35	7:0		2	5	+	+	+	+	+	+	+	–	+	–	nt	+	+	–	ERV/PPZA2	yes	no
gt-36	0:2			2	+	+	+	+	+	+	+	+	+	–	+	+	+	–	ERV/PER/PAS	nt	nt
gt-37	1:6	1	6		+	+	+	+	+	+	+	–	+	+	+	–	–	–	ERV/PPZA2/TDI	yes	no
gt-38	1:3	1		3	+	+	+	+	+	+	+	+	+	+	+	–	–	–	ERV/PER/TDI	nt	nt
gt-39	70:1		70	1	+	+	+	+	+	+	+	–	+	+	+	ψ^*e*^	+	–	ERV/PPZA2/TDI	yes	no
gt-40	4:11			15	+	+	+	+	+	+	+	+	+	+	+	+	+	–	ERV/PER/TDC	yes	yes

*^*a*^Selected pathway genes of different alkaloids: + presence of gene; -absence of gene.*

*^*b*^*idtP* detected in the same *F. rubra-E. festucae* symbiotum in the set of the samples within genotype (see Supplementary Table 4, not published data).*

*^*c*^Predicted chemotypes: CC, chanoclavine; ERV, ergovaline; PER, peramine (equivalent to PPZA1, pyrrolopyrazine-1-ones); PPZA-2, pyrrolopyrazine-1, 4-diones; PAS, paspaline TDI, terpendole I; TDC, terpendole C; LTB, lolitrem B.*

*^*d*^Ergovaline and peramine production of *Festuca rubra – Epichloë festucae* symbionts have been determined chemically by Vázquez de Aldana et al. (2020).*

*^*e*^Expected pseudogene (see Supplementary Table 4).*

*^*f*^PER, peramine a pyrrolpyrazine-1-one.*

*nt, not tested; nd, not detected.*

### Alkaloid Genotypes and Predicted Alkaloid Production

The alkaloid gene profiles of different aMLGs with mating types, isolate numbers and predicted and confirmed chemotypes are presented in Table 5 and host information and alkaloid profiles of the *E. festucae* in Supplementary Table 6. Overall, 38 multilocus alkaloid genotypes (aMLGs) and 20 unique chemotypes were identified among the Spanish, Faroese and Finnish *E. festucae* isolates based on the seventeen genetic loci or alleles associated to ergot alkaloid (*EAS*), indole-diterpene (*IDT*), pyrrolpyrazine (*PPZ*), and loline (*LOL*) production (*n* = 198; Figure 1 and Table 5). Common to all isolates was the absence of genes required for the biosynthesis of lolines. Most of the isolates (94,4%, *n* = 187) are predicted to produce at least one end product of EAS, PPZ and/or IDT alkaloid class. Most (90.4%, *n* = 179) of the isolates had all the *EAS* genes targeted, and thus, they are likely to have a functional *EAS* pathway and are predicted to produce ergovaline. About one fifth (16.7%, *n* = 33) of the isolates contained all *PER* markers and they are expected to produce peramine, the pyrrolopyrazine-1-one PPZ-1-ones (Berry et al., 2019). Isolates (75.8%, *n* = 150) that lack the reductase domain, are predicted to make pyrrolopyrazine-1, 4-diones (PPZA2). Chemotypically determined ergovaline and peramine production in the same *F. rubra-E. festucae*-symbiosis revealed that 72.7% (*n* = 11) and 100% (*n* = 10) of the isolates corresponded with the predicted alkaloid profiles, respectively (Table 5; Vázquez de Aldana et al., 2020). Three isolates contained all functional *IDT* genes and, thus, they are predicted to produce lolitrem B. The earlier IDT pathway intermediates paspaline, terpendoles I and C are predicted to be produced by 1.5% (*n* = 3), 43.9% (*n* = 87), and 8.6% (*n* = 17) of the isolates, respectively.

The numbers of isolates within the aMLGs differed largely from unique genotypes (one isolate/aMLG) to common genotypes (2-71 isolates/aMLG) (Table 5). Most of the aMLGs (81.6%) were only located in one region. Altogether, 31.6%, 36.8%, and 50% of the aMLGs were observed in the Faroe Islands, Finland and Spain, respectively.

In Spain, 40.4% of the isolates possessed different aMLGs (*n* = 19) and considerably higher amounts of predicted chemotypes were detected in three Spanish populations compared to northern populations (Figure 1 and Table 5). The observed 13 different chemotypes are predicted to produce different combinations of final products of ergovaline or lolitrem B and their early pathway products and peramine or pyrrolopyrazine-1, 4-diones (Figure 1 and Table 5). All isolates are expected to be toxic to mammals due to presence of all *EAS* and/or *IDT* genes, except one isolate (gt-21) (Table 5). In contrast, the frequency of PER and PPZA-2 chemotypes that potentially deter insect feeding differ among the populations. Majority of the SPLV (88.2%) and SPPOR (85.7%) isolates can be expected to produce peramine (PER), which is the case of 37.5% in SPGD isolates (Table 5). However, 62.5% of SPGD isolates are expected to produce pyrrolpyrazine-1, 4 diones (PPZA-2), which is the case of one isolate in both SPLV and SPPOR. The most common aMLG, g*t-40* (*n* = 15), contained about one third (31.9%) of the Spanish isolates and occurred in all populations (SPGD *n* = 2; SPLV *n* = 10; SPPOR *n* = 3) (Table 5). *Gt-40* was positive for twelve out of thirteen alkaloid markers and is predicted and confirmed to produce ergovaline and peramine and is predicted to produce terpendoles (Table 5). Significant pairwise differentiation levels based on the occurrence of the alkaloid genes were estimated between the Mediterranean oak forest (SPGD) and both dehesa populations SPLV and SPPOR (Phi_*PT*_ = 0.150, *p* = 0.005; Phi_*PT*_ = 0.101, *p* = 0.037, respectively), but not between the dehesa populations of SPLV and SPPOR (Phi_*PT*_ = 0.054, *p* = 0.106) (Supplementary Table 5C).

In the Faroe Islands, one fifth (20%) of the isolates possessed different aMLGs (*n* = 12). Overall, five unique aMLGs and seven common aMLGs with 91.7% of the fungal isolates were present in the region. Several *IDT* genes were absent in the Faroese isolates (Table 5). Most (88.3% and 90%) of the isolates are expected to produce ergovaline and pyrrolopyrazine-1, 4-diones (PPZA-2), respectively (Figure 1 and Table 5). The most common aMLG, *gt-27* (*n* = 30) was observed in half of the Faroese isolates in five populations and tested positive for seven out of thirteen markers including all *EAS* genes and lacked *perAR* marker and all five *IDT* markers (Table 5). Second most common Faroese aMLG, *gt-33* (*n* = 9) contained 15% of Faroese isolates in four populations and tested positive of eight out of thirteen markers including all *EAS* genes and lacked *perAR* marker and all *IDT* genes except *idtG* (Table 5). No significant pairwise differentiation levels based on the occurrence of the alkaloid genes were detected among the Faroese populations in alkaloid gene variation (*p* > 0.05) except between FAS1 and FAS5 (0.147, *p* = 0.039) (Supplementary Table 5C).

In Finland, only 15.4% of the isolates possessed different aMLGs (*n* = 14). Overall, seven unique and seven common aMLGs with 92.3% of the isolates were detected in the region (Table 5). Majority (91.2%, and 87.9%) of the isolates contained all the *EAS* and *IDT* early pathway genes (*idtG, idtQ*, *and idtP*) and they are expected to produce ergovaline and/or terpendole I, respectively (Figure 1 and Table 5). Most (91.2%) of the isolates are predicted to make pyrrolopyrazine-1, 4-diones (PPZA-2) and one isolate peramine (gt-38; Table 5). The most common aMLG, *gt-39* (*n* = 70) was found in 76.9% of Finnish isolates in all six populations (Table 5). The *gt-39* was positive for 11 out of 13 markers, lacked *perAR* marker and *idtJ* gene, and had non-functional *idtF* gene (determined from ten isolates of gt-39). Thus, gt-39 is likely to produce ergovaline due to presence of all five detected EAS genes, pyrrolopyrazine-1, 4-diones (PPZA-2) due to presence of *perA5* and *perAT2* markers and the indole-diterpenes pathway intermediate terpendole I due to presence of *idtG, idtQ and idtP* (Table 5). No significant pairwise differentiation based on the occurrence of the alkaloid genes was detected among the Finnish populations (*p* > 0.05; Supplementary Table 5C).

## Discussion

Our results suggest that the postglacial colonization history of the host grass, *F. rubra*, and predominance of asexual reproduction in the heritable symbiotic *E. festucae* largely determines its genetic structure in Europe. During the most recent ice age, the Pleistocene Epoch, arctic ice sheet advanced south covering large parts of Eurasia and North America in the Northern Hemisphere. Of the regions examined in this study, parts of the Iberian peninsula, remained ice-free whereas ice sheet covered much of the Northern Europe. However, some of the grass populations may have survived on sporadic glacial refugia on mountain peaks, nunataks, in the shores of Norway, Kola peninsula and Faroe Islands. Thus, we assume that most genetic variation among and within the examined *F. rubra* populations is best explained by independent long-distance colonization events and genetic adaptation to the local environment (Bazely et al., 1997; Dirihan et al., 2016; Leinonen et al., 2019; von Cräutlein et al., 2019). Although selection can operate on the fungus and host individually or in concert as a phenotypic unit, in *Epichloë*-*F. rubra* interactions only one fungal genotype is transmitted vertically to seed progeny, promoting stable interaction between the fungal genotype and the host lineage. Similarly to the host grass (von Cräutlein et al., 2019), here we identified three larger regional clusters of *E. festucae* - southern, northeastern, and northwestern European clusters – with genetic diversity reflecting the genetic divergence detected in the host populations. For clarity, we use the same division as in our previous papers describing geographic variation in *Festuca rubra* L. ploidy levels and systemic fungal endophyte frequencies, and the genetic diversity of the host plant (Dirihan et al., 2016; Leinonen et al., 2019; von Cräutlein et al., 2019). Both neutral SSR and adaptive alkaloid gene markers revealed that genetic and genotype diversity of *E. festucae* was highest in Spain, and markedly lower in the Faroe Islands and Finland. These results support our hypothesis that genetic diversity should be highest in ice age refugia and decrease toward the edges of species range.

### Potential Forces Driving Distribution History, Genetic Diversity, and Geographic Differentiation of *Epichloë festucae* Populations

Because the occurrence of the host plant is a prerequisite for the dispersal of associated symbiotic microbes, the genetic diversity of microbe should primarily mirror forces driving the postglacial distribution of the host species and secondarily the microbes, or the host and the microbe in concert as a phenotypic unit. During the last ice age, ending about 20,000 years ago, glaciers extended over much of northern Europe and also over much of Canada and some of the northern United States. Presently, *F*. *rubra* has a broad circumarctic-circumboreal distribution due to its great adaptive ability to colonize new pockets of land exposed from the retreating ice sheet during the postglacial distribution history (Inda et al., 2008; Braun et al., 2020). Taxonomically *F. rubra* is a morphologically variable species complex showing extensive hybridization, polyploidy, as well as phenotypic and genetic variation (Jenkin, 1955; Markgraf-Dannenberg, 1980; Ainscough et al., 1986; Aiken and Fedak, 1992; de la Fuente et al., 2001; Catalan et al., 2004; Catalan, 2006; Soreng et al., 2017). Thus, distinguishing taxonomic entities as species, subspecies and varieties within the complex is challenging (Saikkonen et al., 2019).

*Epichloë festucae* symbiotic plants can be commonly detected in all three geographic regions examined in this study, but the frequencies of symbiotic plants vary among regions, and populations and habitats within regions irrespective of phenotypic and genetic variation of the host (Dirihan et al., 2016; von Cräutlein et al., 2019). The highest overall endophyte frequencies were found in Spain, where 69% of plants harbored *E. festucae*. In contrast, 36% and 30% of grasses were *Epichloë* symbiotic in Faroe Islands and northern Finland, respectively (Dirihan et al., 2016). Although the fungus is capable of horizontal transmission by sexual spores, contagious spreading within the host populations and long-distance migration among populations or geographic regions appears to be strongly constrained in the Faroe Islands and Finland where the sexual life cycle appears to be extremely rare (Wäli et al., 2007). The present results support this as mating type representation were skewed toward the presence of MTA. Similar geographic patterns of genetic population structures of *F*. *rubra* and *E*. *festucae* suggest that the symbiont has primarily migrated with the host (see von Cräutlein et al., 2019). To date, there has been very little focus on mating type frequency. The recent studies have mainly concentrated on the asexual *Epichloë* species and it appears that only one mating type gene occur in most of the studied species, like *E. festucae* var. *lolii* (Hettiarachchige et al., 2015). In contrast, mating type gene frequency is found in equilibrium in *Epichloë typhina* where sexual stage is active (Bushman et al., 2019).

Here we propose that the classic theory of island biogeography (MacArthur and Wilson, 1967) and the geographic mosaic of coevolution (Thompson, 2005) provide a useful framework to understand distribution, and genetic diversity and geographic differentiation of *E. festucae* populations. Similarly to macro-organisms, a positive species-area relationship has been detected to lead to higher microbial diversity in large and less isolated sampling areas in studies using bacteria diversity in water-filled treeholes (Bell et al., 2005), foliar fungi in birch trees living in fragmented environments (Helander et al., 2007), ectomycorrhizal fungi on “tree islands” (Peay et al., 2007), and soil bacteria and fungi in land-bridge islands as models (Li et al., 2020). Analogously to the prediction that species diversity should reflect “island” size and isolation, we detected highest genetic and genotype diversity of *E. festucae* in Spain which can be treated as a “continent” from where potential *F*. *rubra* and *E*. *festucae* colonists dispersed into Faroe Islands and Fennoscandia. Similarly, the reduction of genetic variation detected in *E. festucae* during the postglacial distribution history appears to be formed by local selection pressures imposing the symbiotum across the examined geographic regions.

In Spain, we found the highest genetic diversity that can be explained by the occurrence of populations near glacial refugia and biodiversity center of fine fescues (Saint Yves, 1930) and occasional sexual reproduction. The two genetically distinct groups of *E*. *festucae* (*Sp1* and *Sp2*) were distributed unevenly in the examined two habitats. The genetically more diverse *Sp2*-group predominated in Mediterranean savannah-like grasslands, dehesa, located higher in altitudes near Salamanca (populations SPLV and SPPOR). *Sp2*-group was detected also in Mediterranean oak forest (population SPGD) to a lesser extent. In contrast, the genetically less diverse *Sp1*-group was prevalent in Mediterranean oak forest and absent in dehesa. The alkaloid gene assemblage differed also among the habitat types and larger variation of alkaloid chemotypes occurred in dehesa compared to Mediterranean oak forest. Nearly all examined fungal lineages had the potential to produce ergot alkaloids with known anti-vertebrate and anti-invertebrate properties as well as peramine or PPZA-2 with anti-invertebrate properties (Ball et al., 1997; Berry et al., 2019; Caradus and Johnson, 2020; Hudson et al., 2021). More than 60% of the fungal isolates from xerophytic forest grasses had genetic potential to produce PPZA-2 and more than 30% of them also terpendoles. Much larger variation of isolates with both anti-vertebrate and -invertebrate properties occurred in dehesa grassland plants and almost all isolates had genetic potential to produce both ergovaline and peramine whereas only two isolates had potential of PPZA-2. Moreover, low genetic differentiation levels between dehesa populations support the similarities in population genetic structure revealed by both SSR and alkaloid markers, as detected in the studies of Arroyo Garcia et al. (2002), Vázquez de Aldana et al. (2010) suggesting adaptation to similar herbivores selection pressures in dehesa habitat. Much lower genetic differentiation was observed among the host grass populations compared to its fungal symbiont populations in Spain, which maybe due the outcrossing of genetically distant host individuals (von Cräutlein et al., 2019). These results suggest that prevailing selection pressures driving distinct prevalence of genetic structures in two different habitats in Spain is operating either on the fungus or fungus-grass genotype combination rather than on the host grass individually, although some variation is observed within dehesa populations (see also Vázquez de Aldana et al., 2010).

In concordance with the presumption that diversity should decrease with the distance to the source regions, some genetic diversity appears to be lost during the colonization of exposed land following retreating ice sheet in North Europe. However, our previous study on the host grass supports the hypothesis that some of the grass individuals may have survived on nunataks in Faroe Islands (Dirihan et al., 2016; von Cräutlein et al., 2019). The present study on *E*. *festucae* in the Faroe Islands do not support the same for the symbiotic fungus. Most Faroese isolates fell into locally adapted *Far*-group, had lost several *IDT* genes and were predicted to produce the same alkaloids across islands. Only three non-local SSR isolates were detected, two isolates from the *Fin*-group in FAS3 population and one isolate from *Sp*2-group in FAS6 population. This suggests that the long-distance co-dispersal of microbes with their hosts occur but not as efficiently into the region as its host *F. rubra* which possess a contact zone of various maternal lineages in the Faroe Islands especially since gene flow from the other locations have been found to be more effective in maritime than inland locations as seeds might arrive by floating and by birds (Saikkonen, 2000; Golan and Pringle, 2017; von Cräutlein et al., 2019). The genetic mismatches between the host and the fungal genotypes can affect infection losses of new plant genotypes during the establishment process and consequently reduce the number of novel fungal genotypes in the region (Saikkonen et al., 2010b; von Cräutlein et al., 2019). Thus, relatively large proportions of non-infected *F. rubra* individuals in Faroese populations may reflect the process of infection losses. In addition, similarly to Spanish populations, the Faroese populations were more differentiated from each other revealed by SSR markers than the Finnish ones, although predominantly two relatively large identical genotypes were present in most of the populations indicating fungal gene flow *via* host seeds among the islands. The observed genetic differentiation of the populations may have been caused by varying selection pressures driving populations in different directions, gene flow from other locations and predominance of vertical transmission of *E*. *festucae* in mainly clonally dispersing *F*. *rubra* in Faroe Islands (Harberd, 1961; Heide, 1990; Saikkonen et al., 1998, 2002; Zhang et al., 2010; Leinonen et al., 2019).

In the species northernmost distribution range in subarctic Finland, the examined *E. festucae* populations present distinct genetic *Fin*-group with relatively low genetic diversity compared to the Southern Spanish populations. Similarly to its host populations of *F. rubra*, one large genotype were observed also in the fungal populations indicating longevity and expansion of local host-fungus genotypes (von Cräutlein et al., 2019). *F. rubra* clones have been detected to be centuries old and occupying large areas (Harberd, 1961) as selection favors the presence of one dominant genotype in a clonal population (Milgroom, 1996). Only two non-local isolates were found in the region, even though relatively high frequencies of non-local host cpDNA haplotypes were observed in the mostly clonal host grass populations (von Cräutlein et al., 2019), which can be due to reproductive differences between the species (Sullivan and Faeth, 2004). Non-local plants can have reduced probability to flower in northern latitudes preventing vertical transmission of non-local *E. festucae* strains (Leinonen et al., 2019). Moreover, infections can be lost by long-distance seed dispersers during the establishment process because infected plants may have a lower fitness compared to uninfected plants especially in harsh conditions (Leinonen et al., 2019). In contrast to Spanish populations with genetically distinct habitat-specific groups, no differentiation was observed among populations divided in two habitats, meadows and riverbanks, in Finland, which may due to efficient local expansion of dominant host-fungus genotypes.

### Alkaloid Production

The alkaloid genotyping has proven to be very powerful to identify potential bioactive alkaloids produced by populations of *Epichloë* (Takach et al., 2012; Charlton et al., 2014; Young et al., 2014; Shymanovich et al., 2017). Genome sequencing of the first two *E. festucae* isolates and other related *Epichloë* species revealed considerable diversity within the genus based on the presence or absence of the alkaloid genes (Schardl et al., 2013a,b,c, 2014; Winter et al., 2018). In the current study, 20 unique chemotypes were predicted from the 198 isolates. These chemotypes ranged from individual alkaloids (e.g., chanoclavine, CC, paspaline, PAS, or the pyrrolopyrazine-1, 4-diones, PZZA-2), to more complex chemotypes representing up to three different classes of alkaloids. Sequencing of genetic loci associated with each alkaloid has revealed repetitive AT-rich transposable elements are associated with alkaloid diversity. In some cases, such as that of the pyrrolopyrazine *perA* gene, transposable elements have disrupted the gene causing the loss of the reductase domain, which results in production of pyrrolopyrazine-1, 4-diones rather than the expected peramine (Schardl et al., 2013b,c; Berry et al., 2015, 2019). In addition, the ergot alkaloid and indole-diterpene loci are located in what appears to be unstable AT-rich regions of the genome in the subtelomere region. Interestingly, we observed in each Spanish population one isolate able to produce lolitrem B. To our knowledge, the production of lolitrem B is rarely observed in *F. rubra-E. festucae* symbiotum and only mentioned in Young et al. (2009). The ergot alkaloid, ergovaline, and the pyrrolopyrazines, peramine and pyrrolopyrazine-1, 4-diones, were most commonly observed within the populations. However, isolates tested for ergovaline and peramine in a previous study (Vázquez de Aldana et al., 2020) did not consistently detect ergovaline when expected. As explanation, the ergovaline pathway has been reported to be silent, with no gene expression observed (Schardl et al., 2013b,c; Charlton et al., 2014; Young et al., 2015). Sequencing this population of *E. festucae* may provide greater insights into the evolution of these biosynthetic genes. The more limited predicted chemotypes found in Finland and the Faroe Islands versus that of Spain, may be due to selection pressure since the Spanish populations may have an advantage of many different chemotypes.

## Conclusion

Reproductive strategy of *E*. *festucae* and mating type distribution likely explain large part of the differences among geographic regions in genetic diversity among the populations within the regions. Low genetic diversity in Finland and Faroe Islands, and lack of differentiation between distinct habitats in Finland, appears to be attributable to the extremely rare production of sexual structures, detectable as symptoms called “choke disease” on the host inflorescences, and dominance of one mating type. For example, we have intensively monitored *F*. *rubra* populations in northern Finland during the last 20 years but never detected choke disease (Saikkonen et al., 2000, 2010b; Wäli et al., 2007). Furthermore, only a few isolates were heterozygous and/or carrying multiallelic loci suggesting multistrain infections or hybrid origins of the isolates e.g., due to somatic hybridization.

Thus, we assume that postglacial distribution history of the host, founder effect, genetic drift and local adaptation of symbiotum largely explain the detected genetic structure of northern *E*. *festucae* populations. In contrast, although past studies have detected choke disease in less than 1% of *Epichloë* symbiotic *F. rubra* plants in Spain (Zabalgogeazcoa et al., 1999), the high numbers of unique genotypes and presence of both mating types in the Spanish populations suggests that the recombination may have an important role in shaping the population structure. On the other hand, unique genotypes can be trapped for centuries within very diverse host genotypes and only compatible combinations in the newly recombined seeds have survived. Future studies will reveal whether random distribution, founder effects and genetic drift rather than natural selection explain the detected imbalanced mating type ratio and thereby decreased genetic and chemotypic diversity of *E*. *festucae* in northern Europe.

## Data Availability Statement

The datasets presented in this study can be found in Supplementary Tables 2, 6, and the name of the online repository and the accession numbers can be found in Supplementary Table 3.

## Author Contributions

MH and KS designed the collection of the data and performed the sampling. MC, HK, CY, and PL designed the genetic study. MC genotyped the isolates and analyzed the data. CY detected and confirmed the alkaloid profiles. MC and KS wrote the manuscript with contributions by all authors.

## Conflict of Interest

CY was employed by the Noble Research Institute, LLC, United States. The remaining authors declare that the research was conducted in the absence of any commercial or financial relationships that could be construed as a potential conflict of interest.

## Publisher’s Note

All claims expressed in this article are solely those of the authors and do not necessarily represent those of their affiliated organizations, or those of the publisher, the editors and the reviewers. Any product that may be evaluated in this article, or claim that may be made by its manufacturer, is not guaranteed or endorsed by the publisher.
